# Adjustment of the dynamic weight distribution as a sensitive parameter for diagnosis of postural alteration in a rodent model of vestibular deficit

**DOI:** 10.1371/journal.pone.0187472

**Published:** 2017-11-07

**Authors:** Brahim Tighilet, David Péricat, Alais Frelat, Yves Cazals, Guillaume Rastoldo, Florent Boyer, Olivier Dumas, Christian Chabbert

**Affiliations:** Aix Marseille Université, CNRS, Integrative and Adaptative Neurosciences UMR 7260, Team Pathophysiology and Therapy of Vestibular Disorders, Marseille, France; Tokyo Medical and Dental University, JAPAN

## Abstract

Vestibular disorders, by inducing significant posturo-locomotor and cognitive disorders, can significantly impair the most basic tasks of everyday life. Their precise diagnosis is essential to implement appropriate therapeutic countermeasures. Monitoring their evolution is also very important to validate or, on the contrary, to adapt the undertaken therapeutic actions. To date, the diagnosis methods of posturo-locomotor impairments are restricted to examinations that most often lack sensitivity and precision. In the present work we studied the alterations of the dynamic weight distribution in a rodent model of sudden and complete unilateral vestibular loss. We used a system of force sensors connected to a data analysis system to quantify in real time and in an automated way the weight bearing of the animal on the ground. We show here that sudden, unilateral, complete and permanent loss of the vestibular inputs causes a severe alteration of the dynamic ground weight distribution of vestibulo lesioned rodents. Characteristics of alterations in the dynamic weight distribution vary over time and follow the sequence of appearance and disappearance of the various symptoms that compose the vestibular syndrome. This study reveals for the first time that dynamic weight bearing is a very sensitive parameter for evaluating posturo-locomotor function impairment. Associated with more classical vestibular examinations, this paradigm can considerably enrich the methods for assessing and monitoring vestibular disorders. Systematic application of this type of evaluation to the dizzy or unstable patient could improve the detection of vestibular deficits and allow predicting better their impact on posture and walk. Thus it could also allow a better follow-up of the therapeutic approaches for rehabilitating gait and balance.

## Introduction

Through the operation of its diverse sensory receptors, the vestibule continually provides information about the position of the head in space and on the slightest accelerations to which it is subjected. Central integration of the vestibular inputs with those of vision and proprioception allows the brain to have in real time, accurate information on the position of our body in space and on our degree of interaction with the environment. In turn this information allows setting appropriate motor responses to maintain static and dynamic balance through coordination of the reflexes of equilibration of the muscles from body, eyes, neck and head [1,2,3].

**Fig 1:**
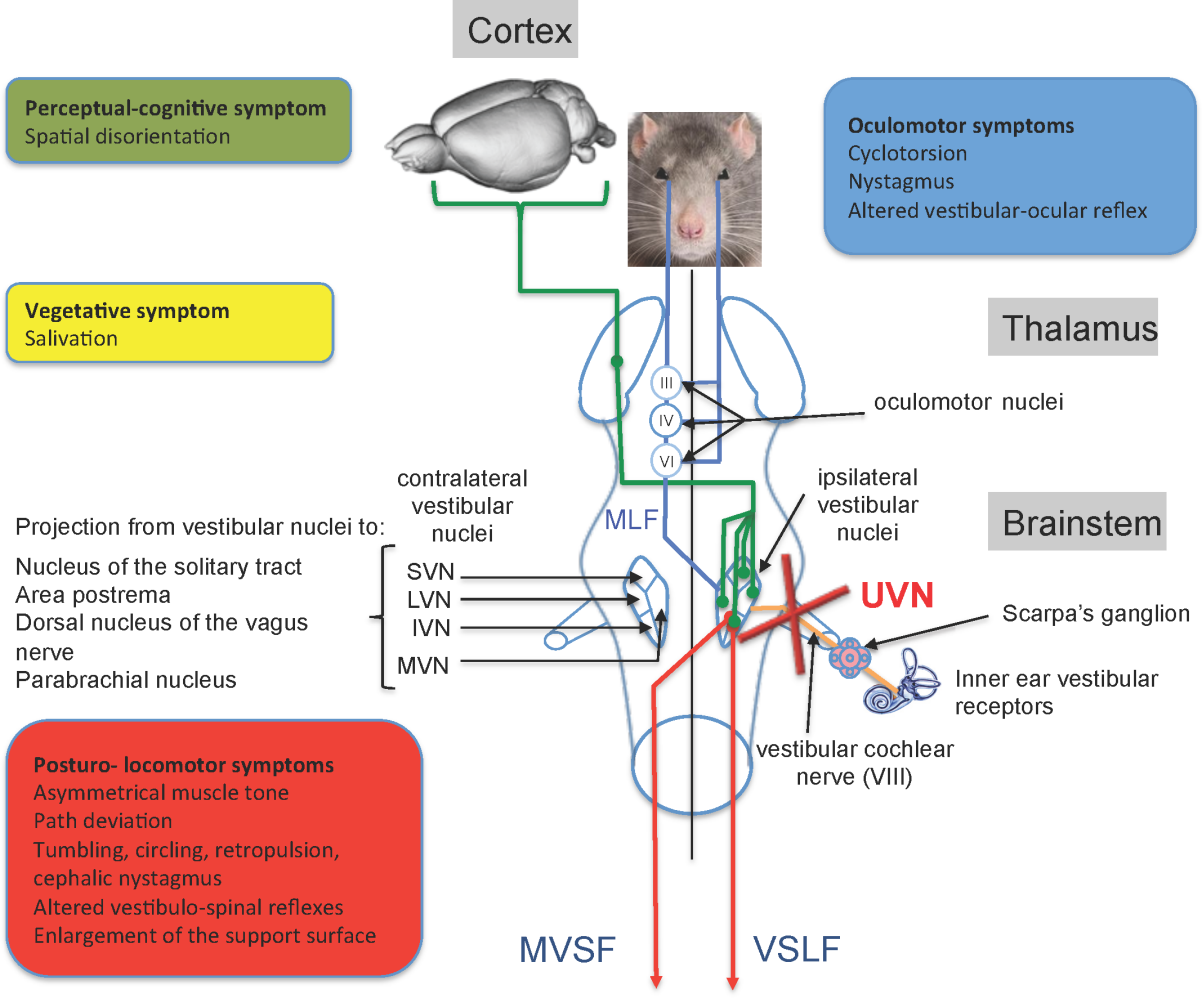
Anatomo-functional organization of the central vestibular system and functional consequences of the unilateral vestibular neurectomy. The vestibular nerve contacts the sensory cells within the vestibular endorgans (semicircular canals, utricle and saccule) and projects ipsilaterally on four vestibular nuclei (VN): the medial (MVN), inferior VN (IVN), lateral (LVN) and superior (SVN) vestibular nuclei. The VNs are located in the dorso-lateral part of the bulbo-protuberantial junction of the brainstem under the floor of the IV^th^ ventricle. They form the first relay of the vestibular-ocular, vestibulospinal and vestibulo-vegetative reflexes and the vestibule-cortical ascending pathways involved in spatial orientation. VNs are the origin of pre-motor messages (control of the ocular and somatic musculature) involved in the regulation of the posture and the stabilization of the gaze during head movements. They project, through the medial longitudinal fasciculus (MLF) on oculomotor nuclei (oculomotor nucleus III, cochlear nucleus or abducens IV) whose motoneurons control the eye muscles to produce compensatory reactions of the eye for stabilizing the images on the retina during head movements. Vestibulo-spinal projections, originating from the ipsilateral LVN, reach all medullary stages via the lateral vestibulospinal fasciculus (VSLF). Contralateral projections median of the median, inferior and lateral VN constitute the median vestibulospinal fasciculus (MVSF) and control the motoneurons of the neck and the upper part of the body axis. VNs receive cerebellar, medullary (proprioceptive) and visual (optokinetic) afferents from contralateral vestibular nuclei and cortical areas. In Human, several cortical areas are involved in the reception of multisensorial vestibular messages (3a area 2v area, parietal-insular vestibular cortex) running through the thalamo-cortical fasciculus. In rodents, vestibular input to the cortex is widespread, affecting many functionally different areas. Some of these areas are homologous with primate vestibular maps (e.g., cingulate and somatosensory cortex), whereas others seem to be specific to rodents (e.g., medial prefrontal cortex). Given this anatomical-functional organization of the vestibular system and its projection targets, unilateral vestibular nerve section induces a quadruple syndrome: posture-locomotor (blue), oculomotor (green), vegetative (pink) and perceptive-cognitive (red). Adapted from [6].

Sudden alteration of the sensory inputs arising from peripheral vestibular sensory and/or neural elements evokes typical vestibular symptoms characterized by a cascade of functional disorders that includes postural imbalance at rest and during movement, spontaneous nystagmus and oscillopsia, associated to cognitive and neurovegetative disorders. These vestibular disorders occur through alteration of the vestibulo spinal, vestibulo oculomotor, vestibulo cerebellar and cortical reflexes [3,4,5,6] (Fig 1).

Current examination of the dizzy or unstable patient generally involves a combined analysis of alterations affecting these three reflex axes. To date, posturographic tests rely on the calculation of the impact of the three main inputs involved in the equilibrium function (vision, vestibular function and proprioception), on the performance of this static equilibrium in terms of surface area, accelerations and displacement of the pressure centre, and also on the energy spent to maintain a static posture under conditions involving or not, vision and proprioception. To our knowledge, there is no static or dynamic platform system currently dedicated to the clinical posturography allowing analysis of the dynamic posture during displacement. The term "dynamic testing” claimed by some manufacturers relies on the platform movement rather than on the analysis of the posture during a voluntary walking activity. Other systems perform kinematic analysis of the body segments or analysis by Wi-Fi inertial sensors of some body segments, without using pressure sensors.

With the aim to identify new parameters for dynamic and sensitive assessment of the evolution of postural control and balance during movement, we studied the characteristics of alterations of the dynamic weight distribution in a rodent model of vestibular lesion. We quantified in real time and in an automated manner, the distribution of the animal weight on the ground, by means of force sensors connected to a data analysis system. This parameter of weight distribution is important as it provides information on animal postural balance, a function that is altered in the vestibulo-lesioned animal. We quantified for the first time the severe alteration of the dynamic ground weight distribution caused by a sudden, unilateral and complete loss of vestibular input. This parameter, which can be assimilated to a biomarker of postural disorder and balance, could be useful in the diagnosis of the vestibular syndrome in human.

## Materials and methods

### Ethics statement

All experiments were carried out in strict accordance with the National Institute of Health Guide for Care and Use of Laboratory Animals (NIH Publication n° 80–23 revised 1996 for the UK Animals -Scientific Procedures- Act 1986 and associated guidelines, or the Policy on Ethics approved by the Society for Neuroscience in November 1989, and amended in November 1993) and under the veterinary and National Ethical Committee supervision (French Agriculture Ministry Authorization: B13-055-25).). Present study was specifically approved by Neurosciences Ethic Committee N°71 from the French National Committee of animal experimentation.

### Animals

Experiments were performed on 11 Long Evans male rats 10–12 weeks old (250/350g) originating from our own breeding from parents arising from Charles River (St Germain sur l’Arbresle, France). Animals were housed in a large confined space with 12h-12h diurnal light variations with free access to water and food. Animals were housed at the Fédération 3C (Centre Saint-Charles, Aix-Marseille University) animal facility.

### Anaesthesia

Animals were anesthetized with a mixture of ketamine 1000 (Virbac; 100 mg/kg, i.p) / medetomidine (Domitor® Orion Pharma 0.2 mg/kg, i.p.). In case of awaking signs additional half dose was provided.

### Surgical approach

All operations were performed on left ears. Access to the vestibular nerve was achieved through the tympanic bulla approach [6]. After shaving the area of interest, animals were positioned on the ¾ back (half the way between lateral and dorsal decubitus). The tympanic bulla was located by groping and incised with a pair of scissors. Dissection was conducted until the muscular planes appear and spreader and paper clips were placed. We then proceeded to the partition of the muscular planes until exposing of the tympanic bulla. The tympanic bulla was widely drilled to expose the stapedian artery and the promontory of the cochlea. The cochlear promontory was perforated at several points using a cutter. Using a 25G bevelled needle inserted into one of the perforations, a leverage movement was performed to detach the promontory and expose the cochlea. After boring the vestiges of the promontory as close as possible to the stapedian artery, the cochlea was removed by pulling with the needle. Remains of the outer bony portion of the cochlea were carefully removed with a fine clip. At this level, the cochlear nerve became apparent. We enlarged the meatus of the cochlear nerve by trimming the bone with a micro needle, then aspirated the 8^th^ cranial nerve with the surgical suction putting the nerve in slight tension in order to cut the entire nerve as close as possible to the brain stem with the needle. The wound was closed using a stapler. Before awakening the animal by intraperitoneal injection of Antisedan® (Orion-Pharma 1mg/kg), a solution of Ringer Lactate (Virbac, 10 ml/kg) was administered subcutaneously in order to alleviate the dehydration resulting from the inability of the animal to drink normally as a result of the injury. The unilateral vestibular neurectomy (UVN) was attested through appearance of the classical postural, locomotor and occulomotor deficits immediately after surgery [6]. The whole surgical procedure was achieved between 20 and 40 min. Upon waking all animals exhibited a severe vestibular syndrome composed of both a tumbling behaviour and spontaneous nystagmus that confirmed the achievement of the unilateral vestibular neurectomy (UNV). The animals received antibiotics for 6 days and analgesics for 48 hours following the lesion.

### Analysis device

**Fig 2:**
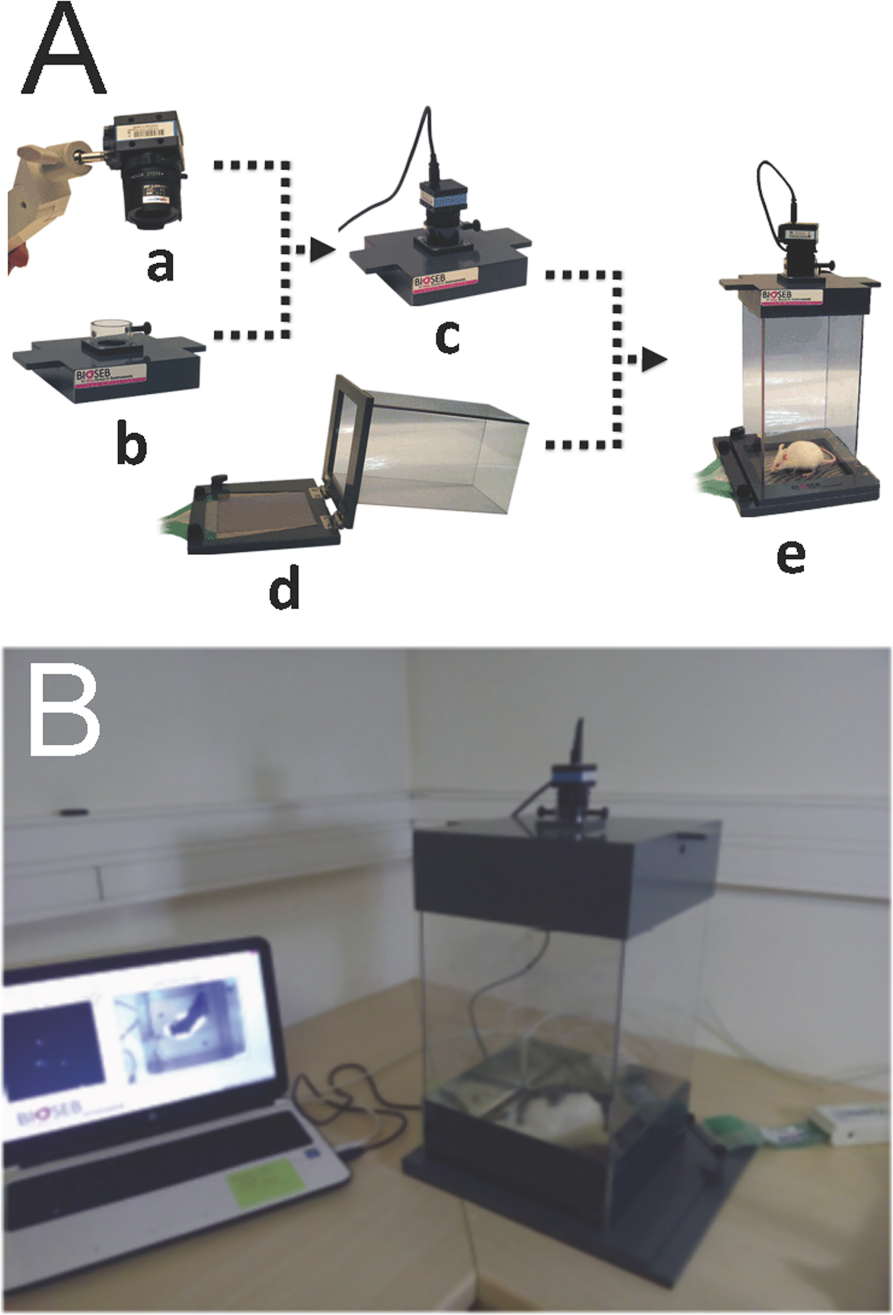
Illustration of the dynamic weigh bearing device used to monitor the dynamic weight distribution in rodents. A: high definition camera (a), coverlid and camera support (b), assembled coverlid (c), force sensors and glass cage (d), assembled system (e). B: Dynamic weigh bearing system in acquisition condition. The rat is free moving in the cage. Its contact points with the captors and the video of its displacement are sent on line to the acquisition software.

**Fig 3:**
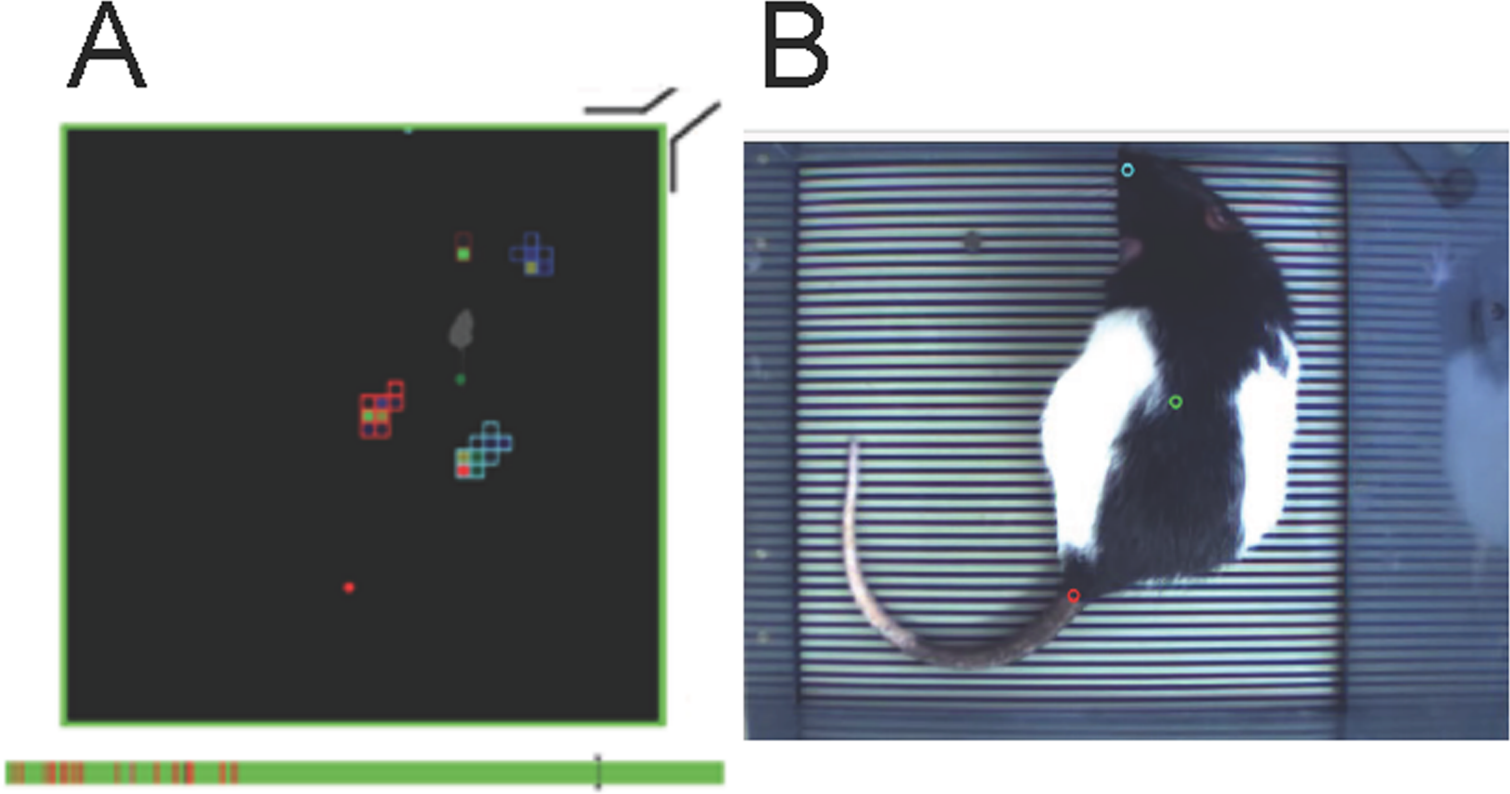
Illustration of the monitoring set up in condition of acquisition. A: a colour is assigned to each area of contact between the animal body and the force sensors. The coloured bar below represents the acquisition time (here 5min). The green corresponds to the time analysed, the red to the non-analysable segments. The grey attachment is a visual marker (representing the electrical connection with the interface) that allows juxtaposition of both the digitized false colour image and the picture of the rat. The grey bar is the cursor of the analysed image. B: Picture taken from the video tracking of the analysed animal.

In order to quantify the postural syndrome following the UVN we used a device (DWB® from BIOSEB) measuring the weight distribution at all contact points of the animal body with the ground (Fig 2). This device consists of a Plexiglas cage (25x25 cm) in which the animal can move freely. The floor of this cage is fully covered with a 2000 force sensors plate. Sensors detect vertical pressure at a frequency of 30 Hertz. The sensors are connected to an electronic interface that converts the current flowing through it into a measure of weight, the whole being connected to a computer. The cage is closed by a lid on which is attached a high definition camera, also connected to the computer through a USB cable (Figs 2 and 3).

### Acquisition in normal situation

**Fig 4:**
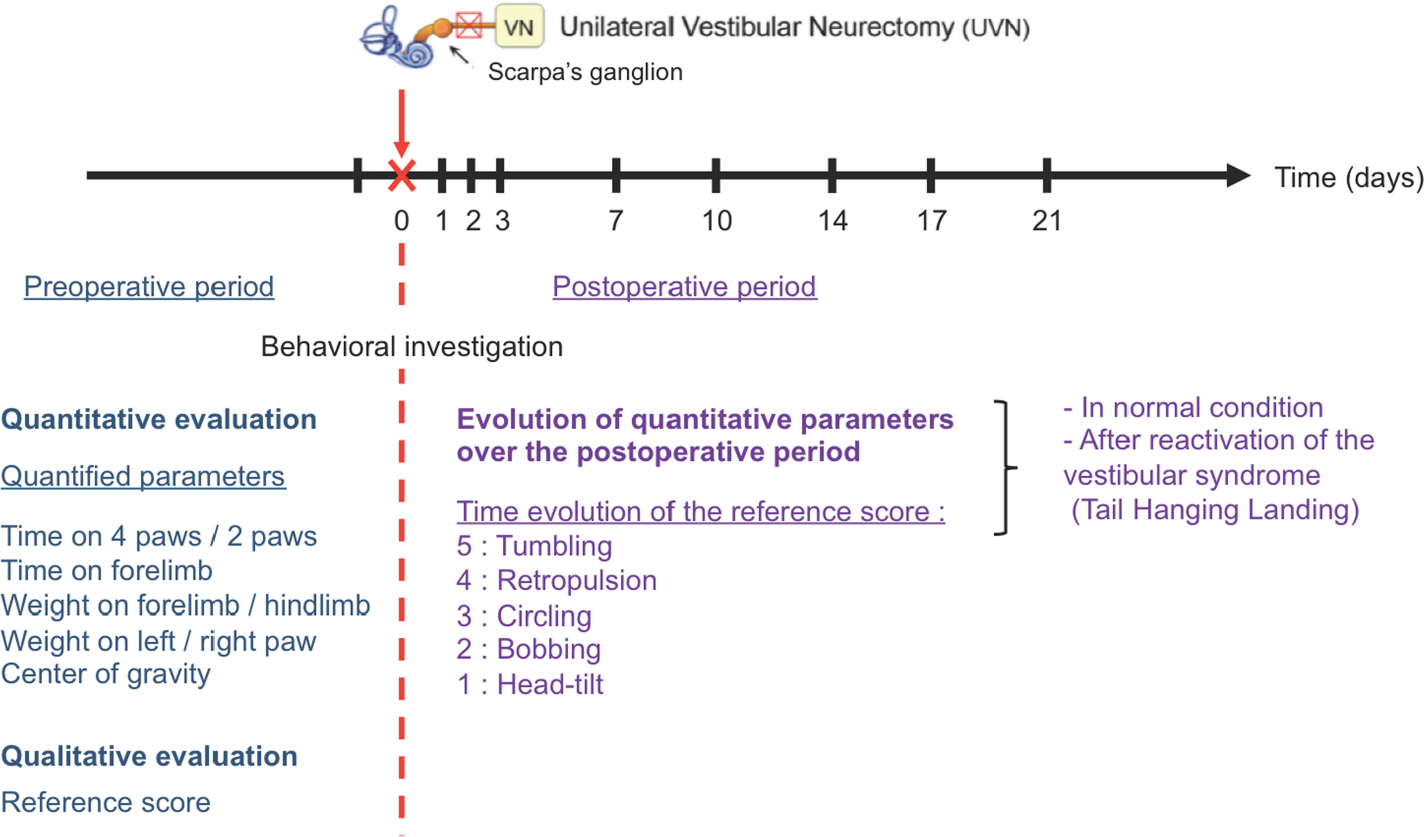
Details of the procedure used to evaluate the alterations of both the posturo locomotor behaviour and the dynamic weigh distribution during the pre-operative and postoperative periods.

Analysis of the various parameters was carried out at different pre- and post-operative times: a first acquisition was made the day before the lesion, serving as a reference value, and then acquisitions were performed at days (D) 1, 2, 3, 7, 10, 17 and 21 post-lesion. For each acquisition session, the animal was placed in the device and moved freely during 5 minutes of recording (Fig 4).

### Data analysis

The analysis consisted in making each group of activated sensors to coincide with the corresponding support paws. The software performed a first automatic analysis of the acquisition file, and then an experimenter visual analysis completed and corrected the initial analysis. The analysis performance depended on the parameters initially chosen. These parameters were kept constant throughout the study duration. The analysis allowed acquiring many parameters. We were interested in the following: a) the time that the animal spent on four, three or two paws during the 5 minutes recording. After a first analysis of the data, we selected the time spent on four and two paws as being most representative of the animal behaviour; b) the time spent on each paw gave indications on the paws the animal uses most often. Since the time spent on the hind paws was always maximal, we chosen to represent the time spent on each of the front paws (left and right) only; This value represented the time during which the left (or right) front paw touched the floor. c) the weight distributed on each paw allowed knowing how the animal distributes its weight on each paw during the acquisition in order to find its equilibrium. We chose to represent on the one hand the weight distribution on the antero-posterior axis (the two front paws in opposition to the two hind paws) and on the second hand the weight distribution on the lateral axis (the front and hind left paws in opposition to the front and hind right paws). In order to compensate for the weight variability of the animals and the variability of the time analysed over the 5 min acquisition, each of the parameters studied was expressed as a percentage of the animal weight on the day of acquisition and as a percentage of the analysed time. Sequences in which the software cannot clearly monitor the paws weight and position where omitted, as well as situation in which the rats put their paw against the wall (Fig 3 bottom red areas). Supporting information is provided at Supplementary information.xls.

### Acquisition after reactivation of the vestibular syndrome

From D1, and for each of the post-lesional delays, a second acquisition of 5 minutes was carried out after subjecting the animal to the tail hanging landing test [7]. This test consisted in taking the animal by the tail and lifting it vertically over a height of about 50 cm (lift duration 2 s; position holding at upper position: 1 s). This test reactivated the vestibular syndrome as a result of otolithic system stimulation and withdrawal of proprioceptive and plantar information.

### Barycenter

Recorded data were used to model the fine postural disruptions of UVN lesioned rats. A recording was done before the UVN which is considered as D0, and subsequent recordings were performed to model the evolution of the weight repartition on the paws through time (D1, 2, 3, 7, 10, 14, and 21). Taking into account that we only measured the weight on the paws, we defined a barycenter-like computation that models weight repartition on the rat paws. Cumulated weight on front and rear right paws in percent of the cumulated weight on the four paws and cumulated weight on front left and front right paws in percent of the cumulated weight on the four paws are respectively represented by the Eqs 1 and 2 (below), with PRP being the percentage on right paws, WFRP, the weight on the front right paw, WRRP, the weight on the rear right paw, PFP, the percentage on the front paws, WFLP, the weight on the front left paw, and TWP, the total weight repartition on the four paws of the animal.

$$PRP=\frac{WFRP+WRRP}{TWP}\times 100$$ (Eq 1)

$$PFP=\frac{WFRP+WFLP}{TWP}\times 100$$ (Eq 2)

Present barycenter-like computation as defined previously is not the representation of the animal centre of mass because it does not take into account the true x and y positions of the animal paws and the weight bearing on other body parts (tail, belly) than the paws. Instead it simply models the left/right and front/hind weight repartitions on paws.

### Behavioural evaluation of the vestibular syndrome

In parallel with the quantitative analysis indicated above, we evaluated qualitatively the vestibular syndrome following UVN using a method recently validated [6]. The vestibular syndrome evoked in the rat after UVN is composed of typical symptoms previously reported in other vestibular disorder models, such as the cat UVN model [8], the rodent models of unilateral excitotoxically-induced transient vestibular deafferentation [9,10,11] or unilateral chemical labyrintectomy [12]. These symptoms, which include tumbling, retropulsion, circling, bobbing and head tilt, are together present in the acute phase and sequentially disappear following specific time courses. The *tumbling* behaviour refers to spontaneous or evoked rotations of the animal along its body axis. This specific parameter (rated 5 in our evaluation scale), only evoked upon most severe vestibular impairments appears immediately following UVN and lasts several hours). *Retropulsion* characterizes backwards movements of the animals. This parameter (rated 4) appears as soon as the rat is able to stand on his four paws and move again, and vanishes after few days. *Circling* (rated 3) describes circular movements of the rats in the horizontal plane that starts with the retropulsion behaviour, but lasts longer, over weeks. *Bobbing* relates to rapid head tilts to the rear and is assimilated to cephalic nystagmus, this behaviour (rated 2) is observed over weeks. H*ead tilt* (rated 1) is an inclination to one side of the head relative to the body axis, it lasts the whole duration of the vestibular syndrome and accompanies even slight and reversible vestibular insults [10]. A rating of zero is given when none of these abnormal behaviours is observed. In the present study, we distinguished the five following states: a first state in which all symptoms are expressed (rated 15) followed by a second stage in which the tumbling has gone (rated 10). A third state in which both the tumbling and the retropulsion behaviours were absent was rated 6. A following state in which tumbling, retropulsion and bobbing were gone was rated 3. Then, two states (rated 2 and 1 respectively) relate to states in which both bobbing and head tilt, or head tilt alone remained.

### Exclusion criteria

Animals were excluded from the study when they exhibited maximum syndrome (score of 15 on the qualitative scale) 48 hours after the lesion, indicating a very strong lesion and very slow functional recovery. We also removed two rats from the study, for which many of the acquisition files were unusable.

### Statistical analysis

For each of the parameters evaluated on the 11 rats sample, the statistical analysis was carried out with the Matlab statistical toolbox software. We performed an analysis of variance (ANOVA with repeated measures with several factors), to test the effect of UVN on the percentage of time spent on two and four paws, the percentage of time spent on the front paws, and the weight distribution (weight percentage on the front and back paws, weight percentage on the left and right paws). Post-hoc analyses were carried out using the Tukey-Kramer multiple comparison test, which is the version of the Tukey correction adapted for comparisons of samples of different sizes. Statistical analysis of the barycenter-like data was performed using repeated measure Anova on each of the two dimensions (weight repartition on the right paws, and weight repartition on the front paws). To this end, we used a Fisher-Snedecor table with α = 0.01%. Here the null hypothesis H0 is the rejection of the mean recording for each days being significantly different and H1, the acceptation of a significant evolution through time. According to the different degrees of freedom, we found 2,8 for the critical value of both dimensions and F-values of 3,491 and 7,056 for the weight repartition on the right paws, and the weight repartition on the front paws respectively. Both F-values being superior to the critical-value, we accepted the H1 hypothesis stating the existence of statistical relevance in the postural evolution for both dimensions with a confidence interval superior than 99%. Anova computations were performed with a python implementation.

## Results

We analysed in a sample of 11 adult Long Evans male rats the consequences of UVN on different parameters: 1- the percentage of time spent on two or four paws, 2- the percentage of time spent on the front paws, and, 3- the right/left and front/back weight distributions 4- the calculation of the barycenter-like measure.

### Comparison of time spent on two and four paws

**Fig 5:**
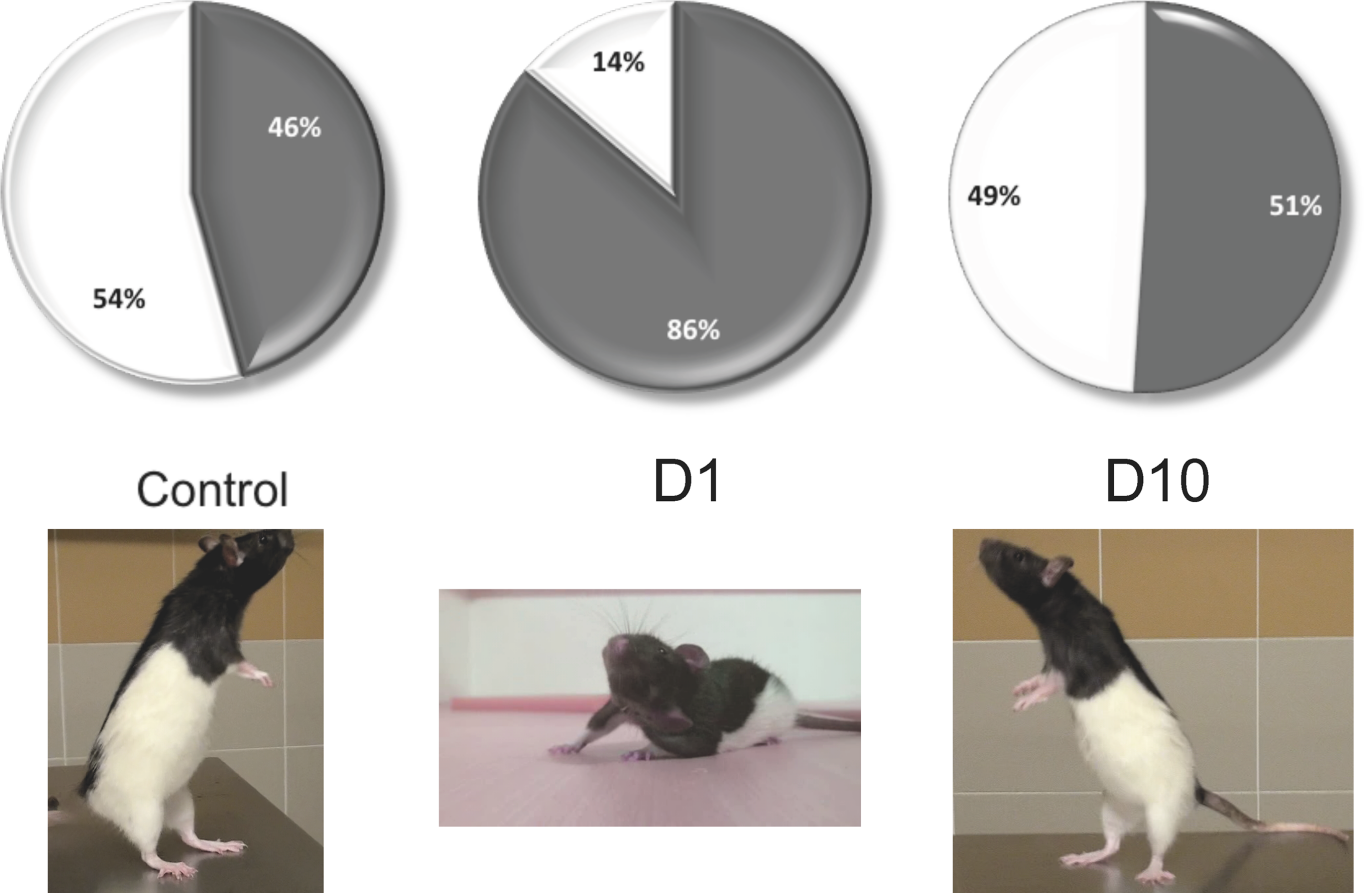
Representation of the time spent on two and four paws in control rats and after 1 and 10 days after UVN. Left: control rats. Middle: 1 day after UVN. This situation corresponds to the acute stage of the vestibular syndrome. Right: 10 days after UVN. This situation corresponds to the compensated stage of the vestibular syndrome. Pictures below illustrate the righting position that characterizes the exploration behaviour (right and left) and the body position characteristic of the acute vestibular syndrome (Middle). At this stage, the rat favours a support surface on the intact side probably due to the increase of the muscular tonus of the paws on the intact side and the simultaneous loss of tonicity on the injured side.

**Fig 6:**
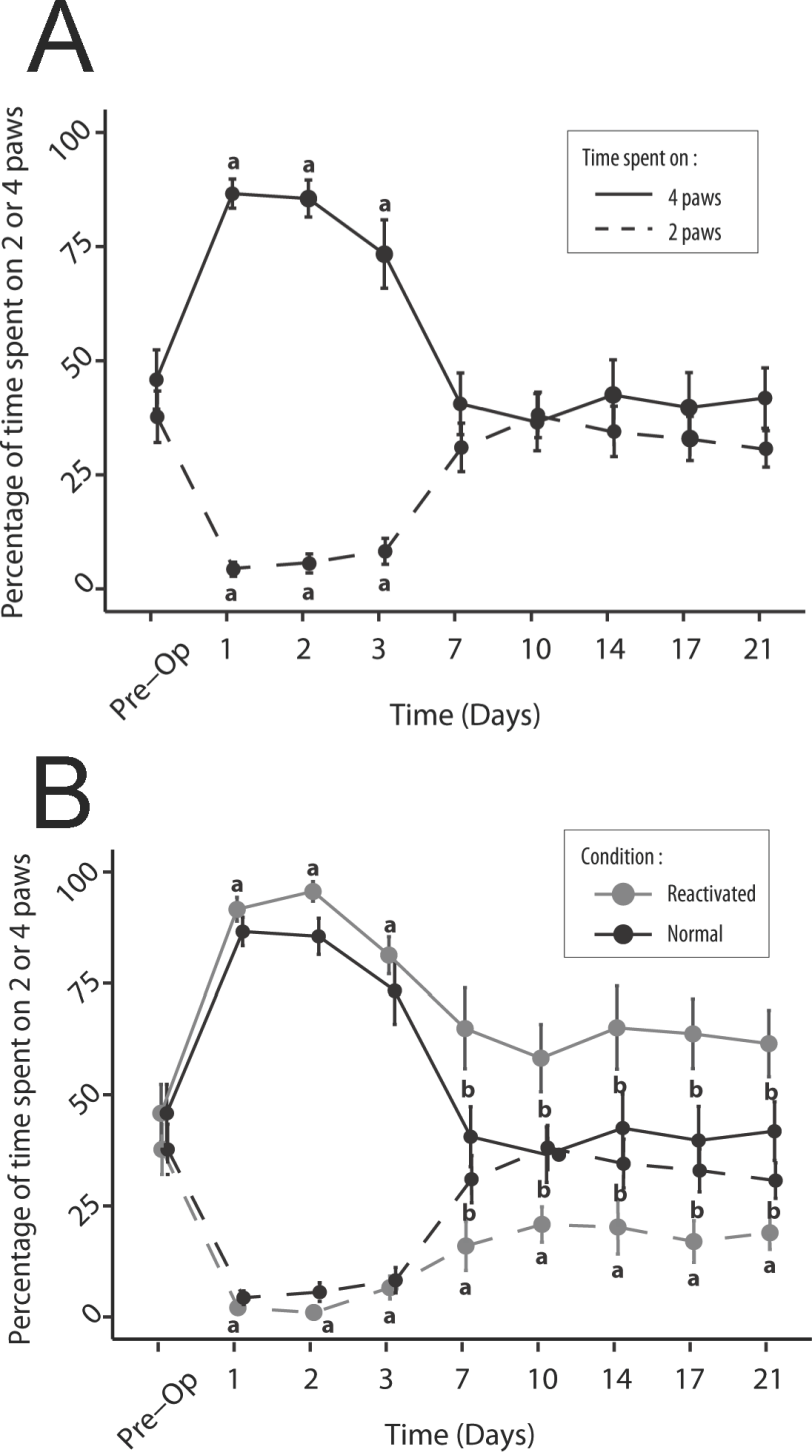
Representation of the time spent on two and four paws for the rats in normal situation and after the UVN. The time spent on two and four paws is represented as a percentage of time compared to the time analysed over the 5 minutes of acquisition. In control situation (A) there is a statistically significant increase in the time spent on four paws and a significant decrease in the time spent on two paws over the first three days after the lesion, relative to the pre-operative condition (a; p <0.05). These changes are followed by a return to preoperative values from D7 in both cases. In reactivated condition (B), there is a statistically significant increase in the time spent on four paws and a significant decrease in the time spent on two paws over the first three days after the lesion, relative to the pre-operative condition (a; p <0.05). Statistically significant change relative to the pre-operative condition is prolonged over the D7-D21 period, only for the time spent on two paws. Over this period, drastic reduction of visual and proprioception inputs induces significant changes (b; p <0.05) in both the time spent on two and four paws relative to the control situation.

Before the lesion, the rats spent the same time on four paws (mean ± SEM = 45.8% ± 6.5) and on two paws (37.7% ± 5.6). After the lesion, the time spent on four paws increased significantly relative to the pre operative time, at the first (86.6% ± 3.2; p<0.001), second (85.5% ± 4.1; p<0.001) and third (73.3% ± 7.5; p<0.03) days, while the time spent on two paws at the same times decreased significantly (4.3% ± 1.6 the first day, p <0.001; 5.6% ± 2.1 the second day, p<0.001; 8.25% ± 2.8 the third day, p<0.001). From Day 7 (D7), these two parameters became equal again and reached values similar to those at pre lesion (Figs 5 and 6A). In conditions of reactivation of the vestibular syndrome, the same tendency as previously described was observed over the first three days after the lesion, with: an increase on the time spent on four paws from D1 to D3 (mean: 89.5% ± 2.0; p<0.001) and a decrease of the time spent on two paws at the same times (mean: 3.2% ± 1.0; p<0.001). From D7, the time spent on four paws decreased again, while the time spent on two paws increased again, although without reaching the preoperative values. Especially, the time spent on two paws remained significantly lower than the preoperative values until D21 after the lesion (mean: 18.6% ± 2.1; p<0.01). From D7 to D21, the time spent on four paws after reactivation of the vestibular syndrome (62.7% ± 3.6) was significantly higher (p<0.05) to the time spent on four paws in normal situation (40.22% ± 3.0). Similarly, the time spent on two paws in reactivated situation (18.6% ±2.1) was significantly lower than the time spent on two paws (33.5% ± 2.2) in normal situation at the same time points (p<0,01) (Fig 6B).

### Comparison of time spent on left and right front paws

**Fig 7:**
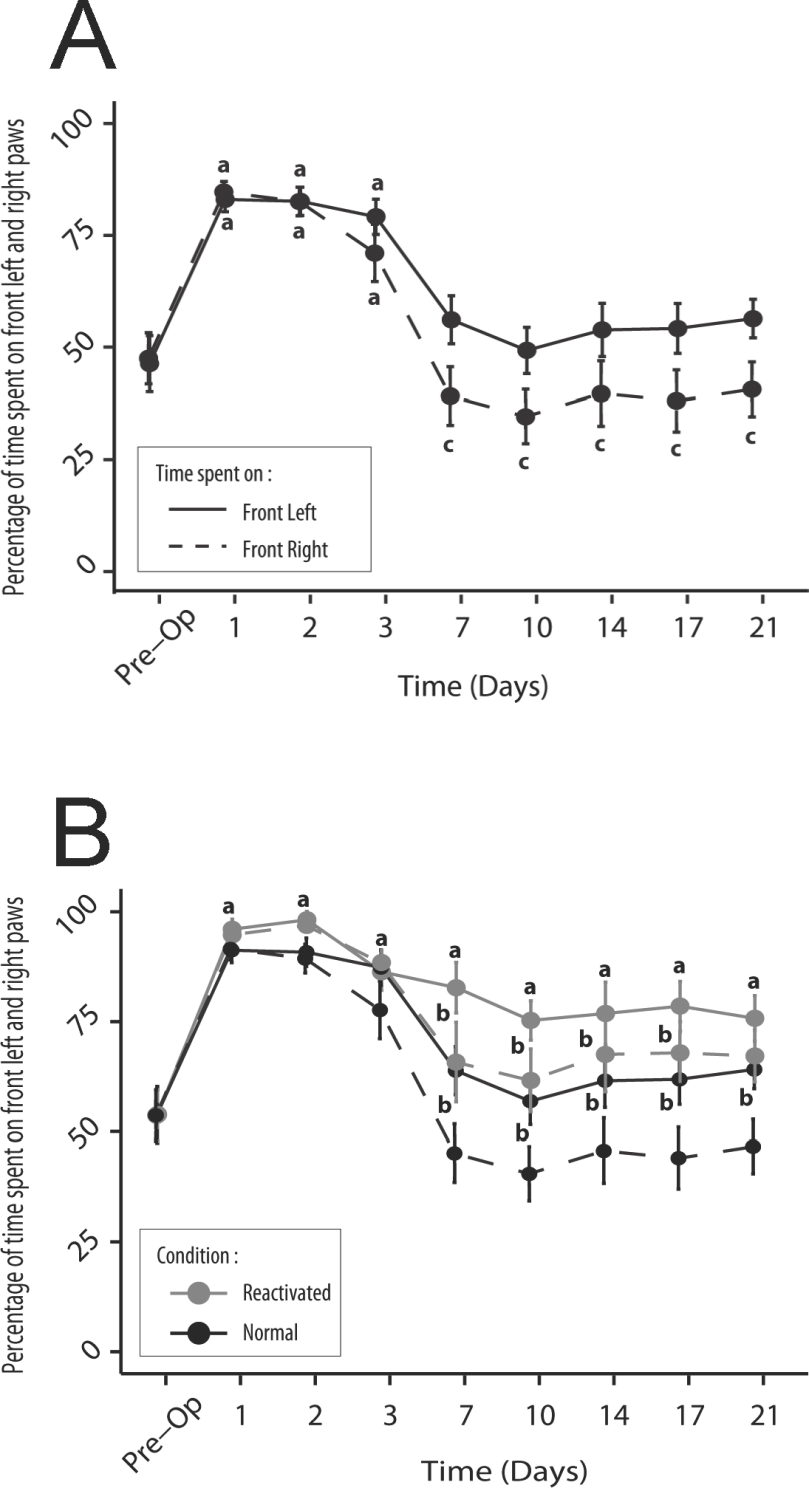
Representation of the time spent on the front right and left paws before and after the UVN. A: In control situation, statistically significant increase in the time spent on the front right and left paws is observed over the first three days after the lesion, relative to the pre-operative condition (a; p<0.05). Over the D7-D21 period, only the time spent on the front left paws remains significantly different relative to the control situation (c; *p*<0,05). In reactivated condition (B), statistically significant change relative to the pre-operative condition is prolonged over the D7-D21 period, only for the time spent on front left paw. Over this period, statistically significant change (b; p<0.05) in both the time spent on the right and left paws relative to the control situation is observed.

Before the vestibular lesion, rats spent as much time on their left front paw (53.8% ± 6.7) as on their right front paw (55.0% ± 5.7, p> 0.05, NS). In the first three days after the lesion, rats spent significantly more time on their left forepaws (mean: 89.1% ± 1.9, p <0.05) and right (86.9% ± 2.7, p <0.05), but always in a left/right balanced manner (p> 0.05, NS). From D7, the time spent on the front paws decreased, but unevenly: rats spend significantly more time on the left front paw ipsilateral to the lesion (on average, from D7 to D21: 61.4% ± 2, 3) than on the right front paw (45.9% ± 2.8, p <0.03) (Fig 7A). After having induced a reactivation of the vestibular syndrome, the time spent on each front leg was again significantly increased during the first three days following the lesion (on average 92.7% ± 1.9 of the time spent on the front left paw, p <0.001, 94.0% ± 1.2 on the right front paw, p <0.001). The time spent on the left front paw remained significantly higher than the preoperative values from D7 to D21 (on average 77.3% ± 2.4, p <0.01), while the time spent on the front right paw decreased to reach values close to pre-lesion values (67.1% ± 3.2, p> 0.05, NS) (Fig 7B).

### Weight distribution along the antero-posterior axis

**Fig 8:**
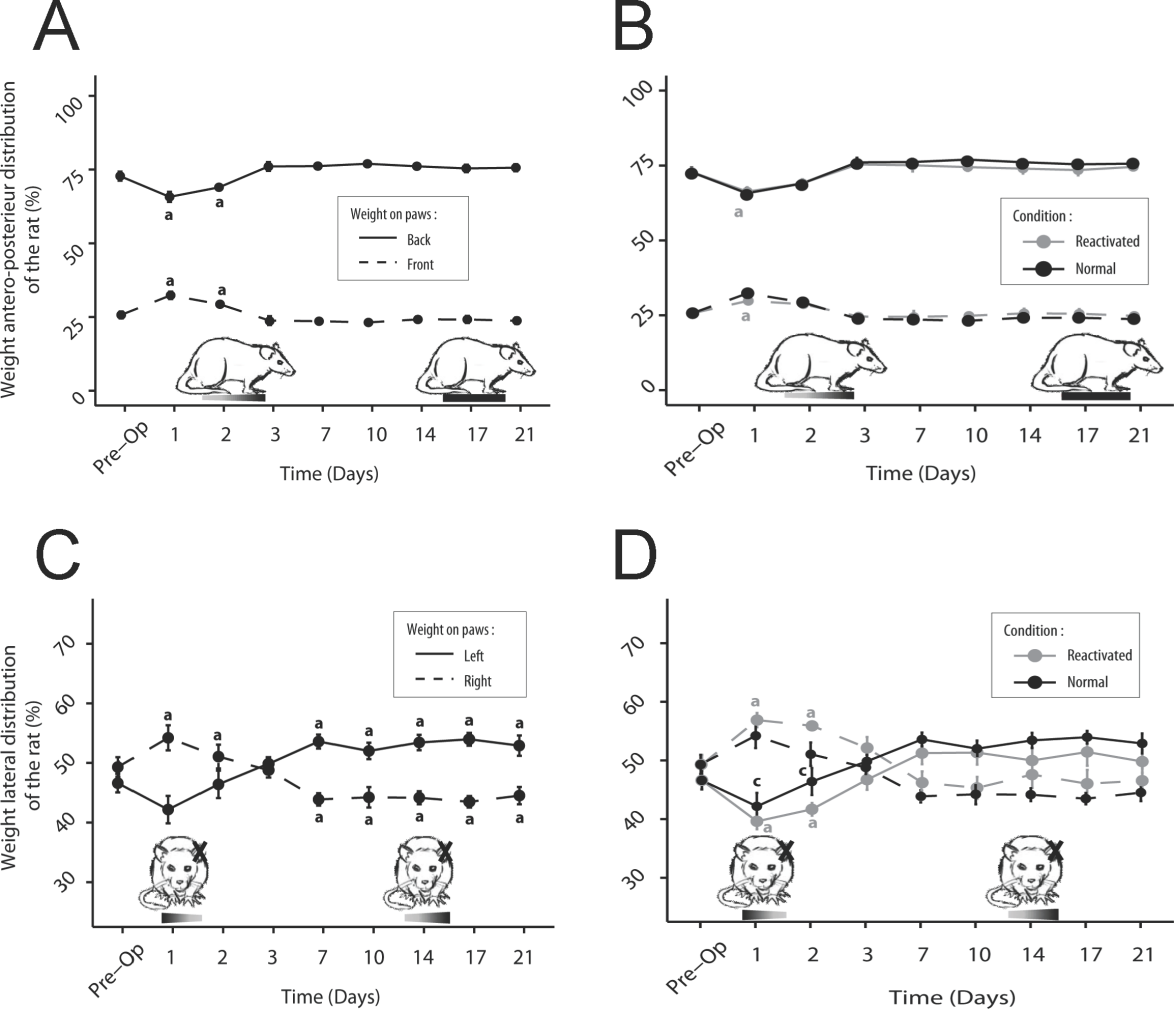
Representation of the weight distribution on each leg before and after UVN. A-B: Antero-posterior axis. Normal situation (A), after reactivation of the vestibular syndrome (B). The rat weight is transferred forward over the first few days, with a statistically significant increase at P1 in the weight applied to the front paws and a simultaneous decrease of the weight on the hind paws relative to pre-operative condition (a; *p*<0,05). Reactivation of the vestibular syndrome does not significantly affect the antero-posterior weight distribution. C-D: Lateral axis. Normal situation (C), after reactivation (D). The rats apply more weight on the contralateral (right) side to the UVN the first two days, with a tendency for simultaneously reducing the weight on the opposite paw. This trend is reversed from P7 and maintained over the 3 weeks period monitored (in both cases: statistically significant difference relative to the pre-operative condition a; *p*<0,05). Reactivation of the syndrome affects only the weight distribution until D2 (c, *p*<0,05).

Control rats have an asymmetric weight distribution on the antero-posterior axis. They distribute their weight at 72.8% ± 1.6 on the hind paws and 25.7% ± 1.2 on their front paws. After UVN, the rats significantly tilted their weight forward. At D1 and D2 the weight applied to the front paws increased (on average 30.8% ± 0.8, p <0.01) while the weight applied to the hind paws decreased (mean: 67.4% ± 1.0, p <0.01). From D3, the weight distribution was identical to that observed before UVN with 23.8% ± 0.4 of the weight applied on the front paws (p> 0.05, NS), compared with 76.1% ± 0, 4 on the hind paws (p> 0.05, NS) (Fig 8A). Reactivation of the syndrome had no effect on this antero-posterior weight distribution at any time (p> 0.05, NS) (Fig 8B).

### Weight distribution along the lateral axis

Healthy rats distribute their weight equally between the left (46.6% ± 1.7) and the right (49.3% ± 1.6) part of their body (p> 0.05, NS). From D1 after UVN, a slight increase in the weight percentage applied on the right side was observed (54.2% ± 2.1, p> 0.05, NS). This was a loss of applied weight on the injured side (42.2% ± 2.3; p> 0.05, NS). The weight distribution between the left and right paws at D1 and D2 differed significantly (p <0.05). This tendency was reversed from D7. Rats mostly distributed their weight to the left until D21 (p <0.001) (Fig 8C). After reactivation, the same trend as in the normal situation was observed. There was a weight tilt to the right (56.40% ± 0.7 of the weight on the right, 40.7% ± 0.9 of the weight on the left, P <0.001) at D1 and D2, followed by a return to equilibrium between left and right at D3 (p> 0.05, NS). From D7, the rat weight slightly tilted to the left (p> 0.05, NS). Again, for this lateral weight distribution there was no significant difference between normal and reactivation conditions (Fig 8D).

### Evolution of barycenter

**Fig 9:**
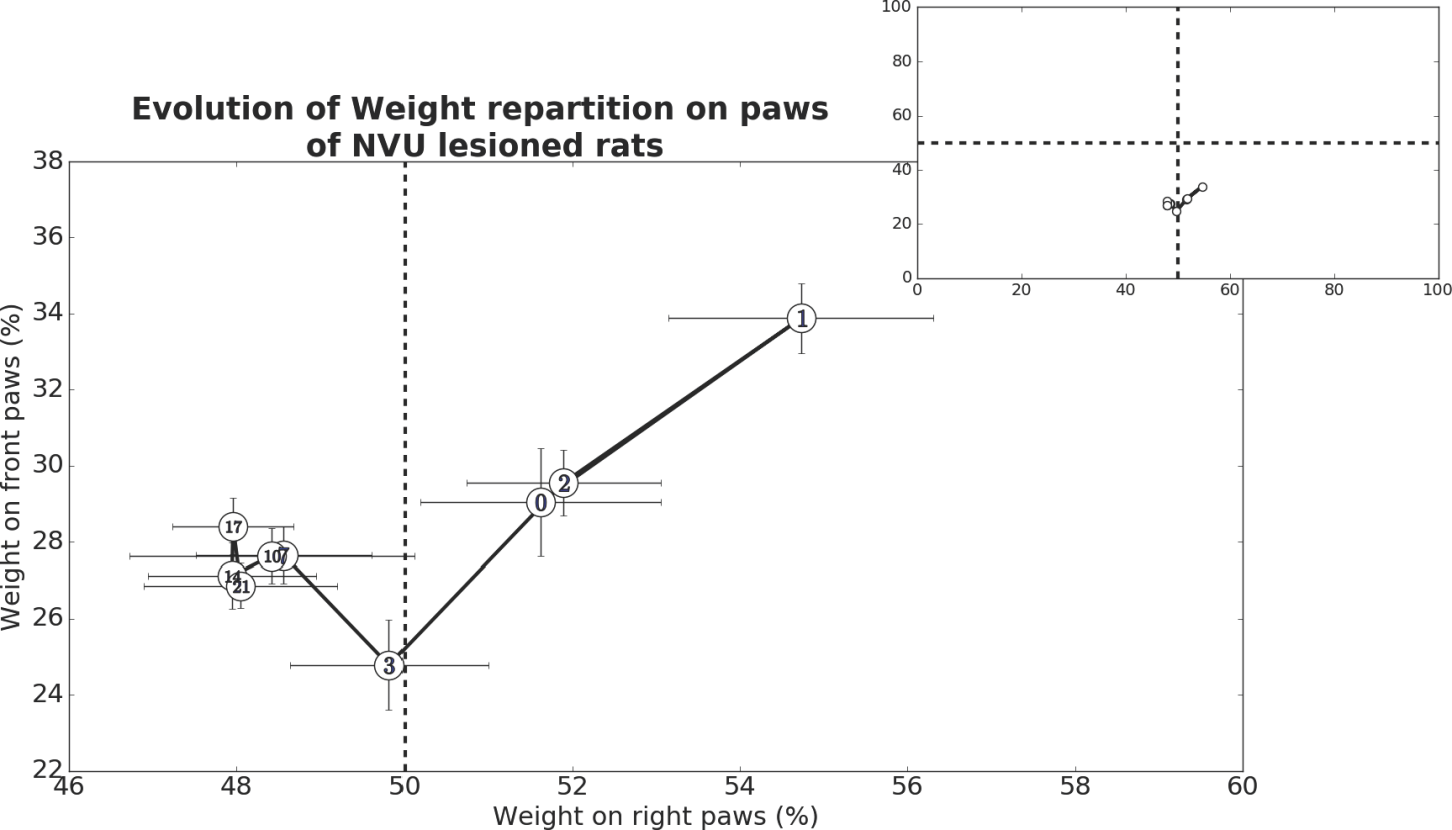
Representation of the barycenter evolution over time following UVN. The abscissa represents the weight percentage on the right paws (cumulated weight on front and rear right paws in percent of the cumulated weight on the four paws), while the ordinate represents the animal weight percentage on the front paws (cumulated weight on front left and front right paws in percent of the cumulated weight on the four paws). The top right panel represents the same dataset than the larger down panel, it only shows the full axis of the figure, and pictures the short range in which our barycentre-like computation varies highlighting the recording of small postural disruptions with the DWB device. At D1, the animal weight shifted on the paws contralateral to the lesion (+3.11% on the right paws of the animal) and on the front paws (+4.83%). Each barycenter-like computation (white numbered dots) are mean barycenter-like calculated from 9 animals and taken from one recording session for each animal.

Measures of the weight distribution over time after UVN were use to estimate the rats barycentre evolution that provides a global representation of the overall postural strategy used at different pre and postlesional delays (Fig 9). For each recording day, we computed the mean weight repartition on the paws for each animal, and then we established the mean between all animals. For the sake of simplicity and statistical relevance, we focused the multiple comparison analysis between the preoperative day (D0), D1 and D21 for the statistical evaluation of early and late postural alterations using a Bonferroni correction. At D1 after UVN, we noticed an acute postural change during which the animals distribute more of their weight on the front paws (p<0.05), and show a tendency to put more of their weights on the right paws (no statistical relevance found), which are on the contra-lateral side of the lesion. Although we did not find statistical differences between the preoperative day and D21 on any dimension, it appears that on the long run, the animals show a tendency to distribute more of their weights on the left paws which is corroborated by the closeness of all means from D7 to D21 on this dimension. Furthermore, we found statistical significant difference for the weight repartition on both dimensions when comparing D1 and D21 with p<0.05 for the weight repartition on the right paws, and p<0.01 for the weight repartition on the front paws. These results indicate that UVN induces both short term and long-term postural changes. Major difference between short and long-term postural modifications is the change in the strategy of weight repartition. With a first tendency to prefer the paws opposite to the lesion (right side), and then a tendency to gradually distribute it more on the paws of the lesioned side (left side). Another difference lies in the animals putting more of their weight on the front paws during short-term postural modifications, which gradually diminish away after few days. Interestingly, the closeness of the means on both dimensions from D7 to D21 is another clue toward long-term postural modifications. Indeed, the stabilization of the animal's posture might be due to a physiological compensation of the sensory deprivation that would take place between D3 and D7. Together these data suggest that compensation of stimuli deprivation occurs in two phases. These results show obvious consistency with the results described above.

### Behavioural evaluation of the vestibular syndrome

**Fig 10:**
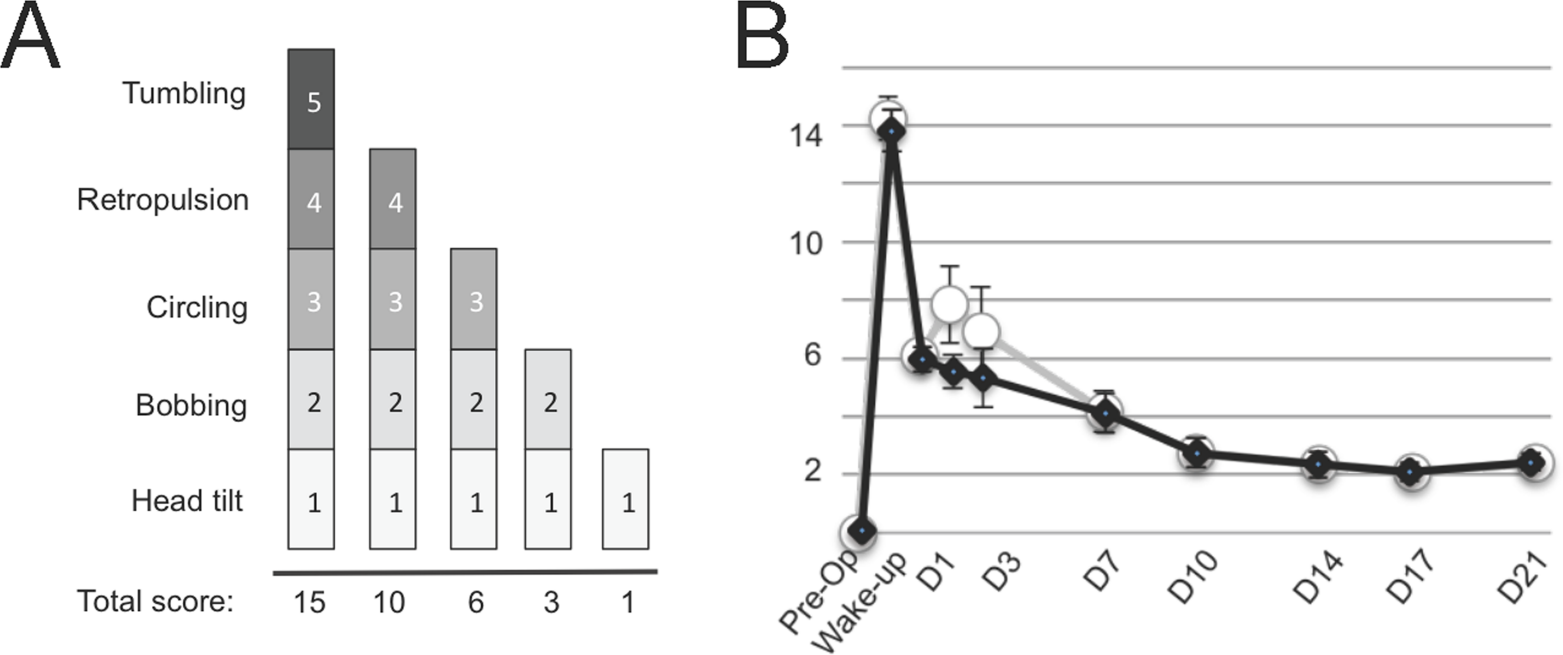
Qualitative assessment of the posturo-locomotor component of the vestibular syndrome. (A) Illustration of the evaluation grid used in present study. A first state in which all symptoms are expressed is rated 15. A second stage in which the tumbling has gone is rated 10. A third state in which both the tumbling and the retropulsion behaviours are absent is rated 6. Then, two states (rated 3 and 1 respectively) related to states in which both bobbing and head tilt, or head tilt alone remained. (B) Illustration of the time course of the behavioural evaluation score involving various posturo-locomotor components of the vestibular syndrome according to the procedure described in Materials and Methods. The expression kinetics of the vestibular syndrome displays different phases: an acute phase of high intensity in the hours following the vestibular lesion (awakening and 4h), an intermediate phase of drastic reduction of the syndrome during the first post-lesion week (D1-D7), and a compensated phase (1–3 weeks), leading to almost complete disappearance of the symptoms. Each data point represents mean vestibular score, with error bars representing sem. Statistically significant difference on the behavioural score is only obtained at D1 and D3. Control (black line and square) and reactivated (white dots and grey line) conditions.

Before the vestibular lesion, all rats are considered healthy, as they do not display any symptoms characterizing vestibular syndrome. As they awaken after the lesion procedure, most rats displayed maximal syndrome (15 on the qualitative scale = tumbling). This characteristic behaviour allows validating the success of the lesion. From D1, the syndrome considerably declined. The rats displayed on average a score of 6/15 on the qualitative scale, mostly corresponding to a circling behaviour. Over time, the consequences of the vestibular damage decreased, though the rats never fully recovered. On D21 all rats still display significant head tilt and some still show cephalic nystagmus. Significant differences (p<0.05) in the qualitative assessment of the vestibular syndrome between normal and syndrome reactivation situations were only observed at two time points within the acute phase (Fig 10).

## Discussion

### Alteration of weight distribution during the vestibular syndrome on the UVN rat model

The present study is the first demonstration that different characteristics of the dynamic weight distribution are significantly modified after unilateral peripheral vestibular impairment. In particular, there is a modification of the time spent and of the weight distribution on the antero-posterior and on the lateral axes. These posturo-locomotor alterations, which significantly impact on the barycenter, match the vestibular syndrome kinetics.

### Time spent and weight distribution along the antero-posterior axis

Healthy rats, in a new environment, display highly developed exploratory behaviour, characterized by half of their time spent on two paws. The characteristic righting behaviour reported as soon as the first uses of the open field in the 1930s [13] allows the rat to explore the environment beyond the confinement zone and potentially anticipate escape behaviour. This original behaviour is accompanied in the healthy rat, by an important exploratory activity. Conversely, the exploratory behaviour is significantly altered in the vestibulo lesioned rat. This is characterized by a significant increase in the time spent on four paws, counterbalanced by a reduction in the time spent on two paws during the first three post-injury days only. These changes are followed by a return to preoperative values from D7. These parameters clearly reflect a strategy aimed at stabilizing the postural balance of the animal. Given that the balance is altered the acute phase of the vestibular syndrome, the animal favours a quadruped postural strategy rather than biped, opposite to what is observed under control conditions. During this phase, the vestibule-lesioned animal also displays a posture intended to increase its contact surface with the ground. The large areas covered by the belly and the tail are drastically increased. The support surface (area between the four paws) is also enlarged [8, 14]. It can be assumed that these adaptive attitudes represent strategies to ensure better postural stability of the animal. This behaviour is normalized as soon as neurobiological processes supporting vestibular compensation are implemented in the vestibular nuclei. The postural asymmetry observed in the acute phase is probably due to the imbalance of excitability between the two homologous vestibular nuclei [15]. This electrophysiological asymmetry between homologous vestibular nuclei is expected to be at the origin of the postural imbalance through inducing an asymmetry of the axial muscles tonus. Conversely, it can be assumed that restoration of the postural balance in the compensated phase results from a return to electrophysiological homeostasis between vestibular nuclei [5, 16]. Analysis of the weight distribution between the front and rear paws shows an asymmetry of weight distribution on the antero-posterior axis of the animal. The weight is distributed significantly more on the hind paws under normal conditions. During the acute phase (D1 to D2), the rats tilt their weight forward. Then, a restoration of the weight distribution identical to that observed before the UVN takes place. These observations validate a loss of equilibrium of the animal and are therefore consistent with the increase of the time spent on four paws.

### Time spent and weight distribution on the lateral axis

As regards of the time spent on the front paws, it is not observed in the rat a tendency to favour one of the front paws during the acute phase (between D1 and D3). This is due to the fact that at these times, the rats spend almost 100% of the time on four paws. They lean as much on the left front paw as on the right front paw. Conversely, as soon as the time spent on four paws decreases (from D7), a strategy favouring a support on the paw ipsilateral to the lesion (left paw) is observed. These data on the animal postural strategy are even more visible when looking at the temporal evolution of the barycenter (equilibrium point) of the animal. One can indeed observe in the rat a displacement of the barycenter on the contralateral side in the first days following the UVN. At this stage, the rat favours a support surface on the intact side probably due to the increase of the muscular tonus of the paws on the intact side and the simultaneous loss of tonicity on the injured side. This behaviour is followed by an inverse trend from post-lesional D7. This indicates that the rat favours first a postural support on the valid side, displaying normal muscle tone at the early postlesional stage as observed in man. The switch of the weight distribution towards the injured side especially visible from P7, but that already starts as soon as P3 may result from the progressive recovery of the muscular tonus on the injured side. This is in correlation with the rebalancing of the spontaneous activity within the vestibular nuclei complex [5]. The persistence of the weight asymmetry over the 3 weeks observation window may also be the consequence of a postural imbalance that is especially visible as the rats start to move again. In the cat, a mirroring effect is observed, with a weight distribution mainly taking place towards the ipsilateral side in the first days following the UVN, and reversing from the 7th post-injury day [17]. The difference with the cat behaviour could be explained by the fact that the rodent exhibits a mixed posture composed of a switch between the two or four paws positions, which conditions its ability to interact with the environment.

### Reactivation of the vestibular syndrome

The procedure of vestibular syndrome reactivation aims at revealing the part of the different sensory modalities involved in the process of vestibular compensation. Under conditions of syndrome reactivation, plantar tactile information’s are totally suppressed while proprioceptive and visual information are significantly diminished. The only system on which the rat can rely to detect information about its position in space is the vestibular system. The vertical lift of the injured animal causes a linear vestibular stimulation in the antigravity direction. This triggers a massive vestibular disorder, probably through restoration and accentuation of the electrophysiological asymmetry between contralateral vestibular nuclei. It can also be assumed that the vestibular disorder induced by this novel dynamic stimulation corresponds to a decompensated situation resulting from the removal of tactilo-plantar, visual and proprioceptive information. Interestingly, especially regarding the time spent on two and four paws, the vestibular compensation is expressed at its peak at D7 and is maintained over the three weeks of our evaluation. It is worthy to note that the decompensating operation does not allow restoring the full syndrome observed on D1-D2. This can be interpreted by the fact that the contribution of the tactile, proprioceptive and visual modalities in the restoration of postural equilibrium is not exclusive, but also depends on remodelling of neuronal networks intrinsically to the vestibular nuclei. During the D1-D3 post-lesional window, a whole battery of markers of neuronal plasticity (cell proliferation, nerve growth factors, factors of inflammation, etc.) is expressed in the deafferented vestibular nuclei. These neuroplasticity mechanisms contribute to remodelling the neuronal networks responsible for the rebalancing excitability between the opposite vestibular nuclei, which is considered in the literature as a key parameter of the vestibular compensation (*vestibulo centric theory*, [5]). We recently demonstrated that during this short and critical time window, major changes take place in the expression of the cation-chloride cotransporter KCC2 (which determine the hyperpolarizing action of GABA) and GABA_A_ receptors in the deafferented vestibular nuclei [16]. These remarkable changes within the vestibular nuclei (VN) strongly suggest that GABA acquires a transient depolaziring action in the VN during the recovery period. This novel and original plasticity mechanism could partly explain how the system returns to electrophysiological homeostasis between the deafferented and intact VN that is essential for the restoration of postural equilibration.

### Behavioural evaluation of the vestibular syndrome vs dynamic weight bearing

Up to date, quantification of the posturo-locomotor syndrome evoked in different animal models of vestibular disorder was achieved through scoring the variations over time of the different symptoms resulting from alterations of the vestibulo-spinal reflex. In present study, such an approach allowed demonstrating that in rodent as in feline, monkey and human, the syndrome is composed of an acute phase in which the syndrome is maximum, an intermediate phase in which the symptoms intensity progressively diminishes and a compensated phase characterized by the almost complete vanishing of the syndrome. Thus, it is possible to account for the fact that certain symptoms (the head inclination in particular) persist for several weeks after the lesion and tend to never fully disappear, conversely to the changes in weigh distribution reported in present study. However, conversely to what was observed by following the dynamic weight distribution, the sole nonparametric “qualitative” analysis of the vestibular syndrome failed to clearly demonstrate an increase in the severity of the vestibular disorder during the syndrome reactivation procedure. Thus, this nonparametric method of evaluation of the vestibular syndrome is not able to account for the contribution of other sensory modalities (vision, tactilo-plantar and proprioceptive information) in the recovery of posturo locomotor function. Vestibular inputs tonically activate the anti-gravity leg muscles during normal posture and locomotion in rodents. Visual, tactilo-plantar and proprioceptive inputs from the paws are very sensitive sensory loops for posturo-locomotor control. The alteration of the dynamic weight distribution in rats that had undergone vestibular neurectomy might reflect a reweighting of the visual, tactile and somatosensory clues controlling balance. Modifying the dynamic weight distribution after vestibular loss may depend on a redistribution of the other sensory inputs. A new sensory selection probably leading to a newly body weight distribution altering the barycenter may be a fast adaptive response to the lesion-induced postural instability. This point is very important because it emphasizes the existence or not of sensory substitutions probably in a differential way as it was observed in humans [18]. This parameter can guide the clinician in the way to re-educate vestibular patients by soliciting one sensory modality rather than another to improve the functional recovery. This observation points out the lack of sensitivity of current evaluation paradigms and the need for more specific evaluation parameters to efficiently monitor and quantify the different aspects of the balance changes that occur during a peripheral vestibular impairment as well as during the vestibular compensation process. The present study reveals for the first time that dynamic weight bearing is a very sensitive parameter for evaluating both posturo-locomotor function impairment and recovery consecutive to UVN. Alteration of the dynamic weight distribution fits with the kinetics of the other components of the vestibular syndrome. Among the undeniable advantages of the automated quantitative analysis vs subjective analysis, is the fact that it minimizes user-dependent errors. It could thus be more adapted to the study of drug effects or the benefits of functional rehabilitation approaches. Together, these observations reveal that variation in dynamic weight distribution is a very sensitive parameter for vestibular lesions and therefore must be considered as a real component of the syndrome. Weight distribution assessment also provides information on the barycentre variations, which is by definition the equilibrium point of the animal. Therefore it brings a more global vision of the animal equilibrium. Combination of qualitative nonparametric and quantitative parametric methods for the evaluation of the vestibular syndrome thus constitutes a real gain enabling us to identify various aspects of the vestibular syndrome.

### Clinical relevance

To our knowledge there is no study comparing the data collected in static or dynamic posturography on the force platform with those collected with devices allowing dynamic analysis during walking. Current explorations of the dizzy or unstable patient allow gathering information to diagnose dysfunction of the vestibulo-ocular pathway (via caloric tests, vestibular head impuls test -VHIT-, kinetic tests on rotating chair), the vestibulo-spinal pathway (with posturography) or to detect any vestibular dysfunction (cortical Vestibular Evoked Myogenic Potential -cVEMP- and ocular VEMP, Echodia® for Meniere and imaging). These various tests also allow monitoring the possible recovery of the vestibule function as well as implementation of the compensation and substitution processes. It is worthy to mention that the VHIT allows topographic localization and quantification of the vestibular defect (on semi-circular canals) and to reveal a possible recovery of the function after deficit. It also allows observing and quantifying the implementation of early saccadic strategy that takes the place of the Vestibulo Ocular Reflex. It has not been demonstrated so far, for example, that observation of any abnormality on the VHIT may be a predictor of fall. One can cite in particular the so-called presbyvestibuly (aging of the vestibular function) which can be revealed on the VHIT. Similarly, for unilateral deficits, VHIT does not provide data that can be correlated to any static or dynamic posturographic data. Conversely, posturography allows monitoring the evolution of the vestibulo-spinal performances in patients, but only in static posture as regards the conventional force platforms. Though present data obtained from quadripedal cannot be fully transferred to humans as postural strategies in man are obviously differs from rodents, one can imagine that the study of the dynamic weight distribution in the dizzy or unstable patient could allow a more documented diagnostic and follow-up, based on paradigms more ecological than those used in existing explorations. It is also to be hoped that strong predictive indicators of the risk of fall could be easily identified through these dynamic measurements, which would also allow widening the indications to the examination of the elderly.

### Conclusion

This is the first report of a survey of a vestibular deficit and its compensation through assessment of the dynamic weight distribution. From an evaluation of the vestibular disorder essentially qualitative, based on subjective measures, we bring by this work a new method of quantitative analysis, based on objective measurements. The work carried out during this study allowed validating the interest and sensitivity of this paradigm for the evaluation of the vestibular disorder and its compensation following a peripheral insult. As the parametric approach provides a better sensitivity for evaluation, we can already anticipate that it would become an essential evaluation tool to diagnose the vestibular syndrome, follow its expression kinetic and test in this time window the effectiveness of antivertigo drugs. In addition, information on the postural strategies adopted after vestibular lesions can guide re-educational approaches to optimize functional restoration. The richness of the data collected thanks to the device for measuring alterations in the dynamic weight distribution (time spent on each paw, weight distribution, barycenter) is likely to further improve the analysis of clinical signs of the vestibular syndrome and optimize their management. Moreover, the use of the dynamic weight-bearing device used in this study could allow identifying new markers of the different types and stages of vestibular lesions and thus allow the identification of novel tools for diagnosis and follow-up of vestibular pathologies.

## Supporting information

S1 File Supporting information is provided at S1_File.xls. (XLS)
